# B Cell Receptor Repertoire Analysis of the CD21^lo^ B Cell Compartment in Healthy Individuals, Patients With Sjögren's Disease, and Patients With Radiographic Axial Spondyloarthritis

**DOI:** 10.1002/eji.202451398

**Published:** 2024-12-20

**Authors:** Rick Wilbrink, Linda van der Weele, Anneke J. P. L. Spoorenberg, Niek de Vries, Ilse T. G. Niewold, Gwenny M. Verstappen, Frans G. M. Kroese

**Affiliations:** ^1^ Department of Rheumatology and Clinical Immunology University of Groningen University Medical Center Groningen Groningen the Netherlands; ^2^ Department of Rheumatology & Clinical Immunology Amsterdam Rheumatology and Immunology Center (ARC) Amsterdam UMC, University of Amsterdam Amsterdam the Netherlands

**Keywords:** B cells, BCR repertoire, CD21^lo^ B cells, radiographic axial spondyloarthritis, Sjögren's disease

## Abstract

B cells with low or absent expression of CD21 (CD21^lo^ B cells) gained attention due to their expansion in the peripheral blood of patients with immune‐mediated, rheumatic diseases. This is not only observed in typical autoimmune diseases like systemic lupus erythematosus and Sjögren's disease (SjD) but also in radiographic axial spondyloarthritis (r‐axSpA), which is considered an autoinflammatory disease. To gain more insight into the origins of the heterogeneous CD21^lo^ B‐cell population, and its relation to the plasmablast (PB) compartment, we profiled the B‐cell‐receptor (BCR) repertoire in CD27^−^ and CD27^+^ fractions of CD21^lo^ B cells and early PBs using next‐generation sequencing. Populations were sorted from peripheral blood of healthy individuals, SjD patients, and r‐axSpA patients (*n* = 10 for each group). In healthy individuals and both patient groups, our findings indicate that CD27^−^CD21^lo^ B cells, which exhibit few mutations in their BCR, may develop into CD27^+^CD21^lo^ B cells and PBs, both marked by considerably more mutations. Given the known expansion of circulating CD27^−^CD21^lo^ B cells in SjD and r‐axSpA patients and clonal relationships with both CD27^+^CD21^lo^ B cells and early PBs, these cells might actively contribute to (pathological) immune responses in rheumatic diseases with autoimmune and/or autoinflammatory characteristics.

## Graphical abstract

This study demonstrates that CD27^−^CD21^lo^ B cells harbor a relatively low frequency of somatic mutations in their BCR compared with CD27^+^CD21^lo^ B cells and early plasmablasts. Clonal relationships were present among all three subsets in both health and disease, indicating that CD27^−^CD21^lo^ B cells actively contribute to immune responses.

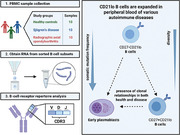

## 1 Introduction

B cells play a crucial role in adaptive immunity, safeguarding individuals against invading pathogens. When exposed to foreign antigens, B cells can become activated and differentiate into plasmablasts (PBs) or plasma cells, generating large quantities of antibodies that bind to and facilitate the elimination of these foreign antigens [1]. During the early stages of B cell development, an extremely polymorphic B cell receptor (BCR) repertoire is generated, allowing the recognition of a wide range of foreign antigens. To this end, a set of variable (V), joining (J), and in the case of the immunoglobulin heavy chain (IGH), also an additional set of diversity (D) genes, rearrange to form the variable part of the heavy and light chain of the BCR, which are unique for a particular B cell [2]. Following B cell activation, the BCR is typically fine‐tuned in germinal centers by a process called affinity maturation. At these anatomical sites, activated B cells divide and undergo somatic hypermutation, aiming at enhancing the affinity of the BCRs for antigens [3]. This process involves the accumulation of mutations in the rearranged germline sequences, often occurring in the complementarity‐determining regions (CDRs) [4]. Especially the CDR3 region plays a crucial role in antigen recognition, as its variability directly influences the specificity and affinity of the BCR. After multiple rounds of cell division and selection within germinal centers, memory B cells with mutated BCR sequences leave the germinal center and may differentiate into PBs or plasma cells [3]. Alternative to this germinal center route, some activated naïve B cells may differentiate directly into PBs through an extrafollicular response, harboring only very few mutations within their BCRs [5, 6]. As a consequence of B cell tolerance, B cells are usually not responding to self‐antigens. However, in the context of autoimmune diseases, the regulatory mechanisms of B cell tolerance are compromised, resulting in the production of autoantibodies, often accompanied by alterations in the distribution of the peripheral B cell compartment [7, 8, 9]. Patients with autoimmune diseases may also exhibit variations in CDR3 length, with both shorter and longer CDR3 lengths observed in patients compared with healthy individuals [10, 11], depending on the specific B cell population being studied (e.g., naïve or isotype‐switched cells).

Over the past decade, B cells characterized by a low expression or absence of CD21 (CD21^lo^ B cells) gained interest due to their expansion in the peripheral blood of patients with autoimmune disorders such as systemic lupus erythematosus (SLE), rheumatoid arthritis (RA) and Sjögren's disease (SjD) [12, 13, 14]. These CD21^lo^ B cells exhibit heterogeneity, encompassing both CD27^−^ and CD27^+^ cell populations. The cell surface protein CD27 is often used to distinguish between naïve and memory B cells in humans [13]. Although most memory B cells indeed express CD27, some isotype‐switched IgD‐negative B cells lack CD27, often referred to as double‐negative B cells [15]. Previous studies showed that at least part of the CD21^lo^ B cells are autoreactive and considered to be anergic, as revealed by the absence of B cell activation marker expression, along with impaired intracellular calcium mobilization, upon BCR simulation [14, 16]. In contrast, the pool of CD21^lo^ B cells may also comprise precursor cells for PBs, as seen after vaccination and in autoimmune conditions [12, 17]. The origin of the various CD21^lo^ B cell subsets, and whether they are derived from germinal centers, extrafollicular responses, or both, is still largely undetermined [12].

Previously, we observed that not only patients with a systemic autoimmune disease characterized by B cell hyperactivity (e.g., SjD) but also patients with axial spondyloarthritis (axSpA) have an elevated frequency of circulating CD27^−^CD21^lo^ B cells compared with healthy individuals [14, 18]. AxSpA is a chronic rheumatic disease primarily affecting the sacroiliac joints and the spine. The disease is considered to be largely an autoinflammatory condition without the involvement of B cells in the pathogenesis [18, 19]. However, culminating evidence indicates that also in this disorder, autoimmunity and B cells may play a role in the disease process [20]. This is for example witnessed by the beneficial effects of B cell depletion therapy with rituximab in r‐axSpA patients, in particular, those who are naïve to anti‐TNF therapy [21]. Moreover, studies have identified autoantibodies, such as those directed against CD74, in patients with axSpA [22, 23].

Given the elusive origin and function of CD21^lo^ B cell subsets in various autoimmune diseases, in this study, we aimed to gain insight into the BCR repertoire of both CD27^−^CD21^lo^ B cells and CD27^+^CD21^lo^ B cells as well as PBs, and the clonal relationships between these subsets, in the context of health and disease [18]. In addition to healthy individuals, we included patients with SjD, a typical humoral autoimmune disease, as well as patients with radiographic axSpA (r‐axSpA), for whom the role of B cells in the disease is less clear. In both diseases, the frequency of CD27^−^CD21^lo^ B cells is increased in peripheral blood, but clonal characteristics of this cell population, and potential relatedness to effector cells, remain unclear. We, therefore, employed next‐generation bulk sequencing to analyze the BCR repertoire of CD27^−^CD21^lo^ B cells, CD27^+^CD21^lo^ B cells, and early PBs (CD20^+^CD27^+^CD38^++^), along with total B cells (CD19^+^CD20^+^) as a reference group. We focused on establishing BCR diversity, the mutational status, usage of IGHV and IGHJ genes, physicochemical properties of antigen‐binding sites, and clonal relationships between the two CD21^lo^ B cell populations and PBs in SjD and r‐axSpA patients in comparison to healthy individuals.

## 2 Results

To gain insight into the BCR repertoire of CD27^−^CD21^lo^ B cells, CD27^+^CD21^lo^ B cells, and early PBs, and clonal relationships among them in health and disease, we sequenced and compared the immunoglobulin repertoires of these populations isolated from peripheral mononuclear cells (PBMCs) of 10 healthy controls (HCs), 10 SjD, and 10 r‐axSpA patients. Our sorting approach included CD19^+^CD20^+^ B cells, enabling focus on relatively early‐stage PBs [24]. Total (CD19^+^CD20^+^) B cells were used as reference and control. One SjD patient was excluded from the study because this patient had an ongoing prosthetic infection. After quality control and removal of duplicate sequences, a dataset was realized that comprised 914,948 unique sequences (Supporting Information File S1, Sheet 4).

### 2.1 CD27^−^CD21^lo^ B Cells Harbor a More Diverse Clonal Repertoire Compared with CD27^+^CD21^lo^ B Cells and Early Stage PBs in both Healthy Individuals and Patients With Rheumatic Disease

First, we searched within each individual for the presence of clonally related sequences within each of the three B cell subsets. CD27^+^CD21^lo^ B cells and early PBs harbored the highest mean percentages (~85% and ~95%, resp.) of clonally related sequences of all analyzed B cell populations (Table S1). This was a consistent finding in all study groups. In CD27^−^CD21^lo^ cells, this proportion was roughly 50%. There were no major differences in percentages of clonally related sequences between HCs, SjD, and r‐axSpA patients.

**FIGURE 1:**
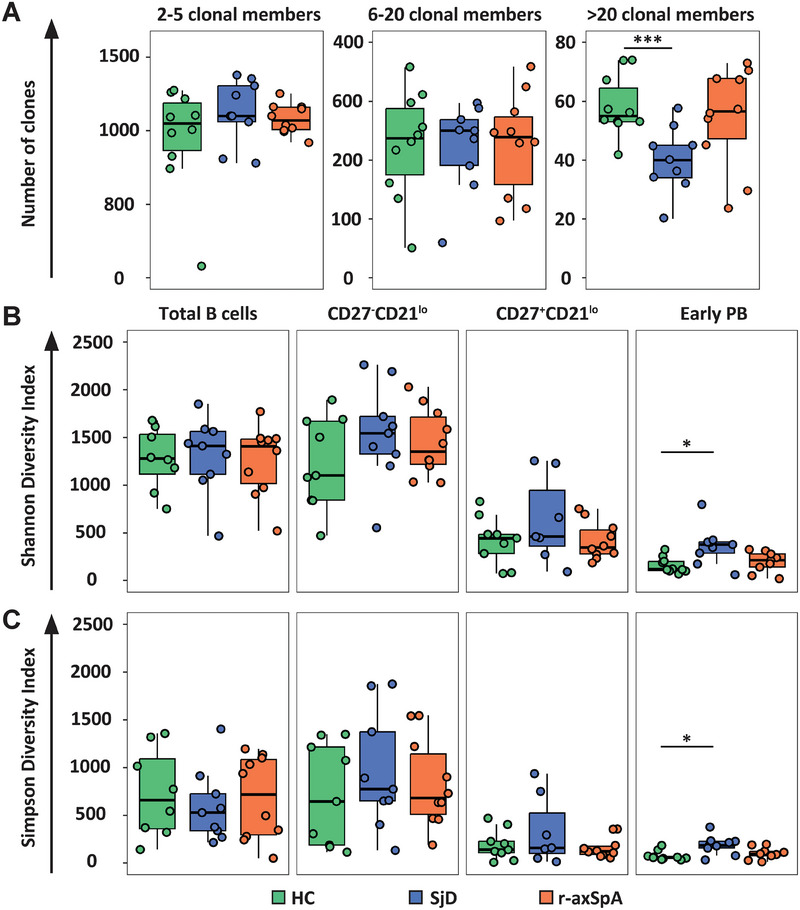
The Repertoire diversity of CD27^−^CD21^lo^B cells, CD27^+^CD21^lo^ B cells, and PBs is largely similar between HCs and patients with SjD or r‐axSpA. (A) Boxplots displaying the number of clones per clonal group size, categorized as low clonality (2–5 clonal members), moderate clonality (6–20 clonal members), and high clonality (>20 clonal members) derived from clonally‐related sequences of CD27^−^CD21^lo^ B cells, CD27^+^CD21^lo^ B cells, and early PBs, shown for healthy controls (HC, *N* = 10), Sjögren's disease (SjD, *N* = 9), and radiographic axial spondyloarthritis (r‐axSpA), *N* = 10). Individual study subject data points correspond to estimated means of (B) Shannon's or (C) Simpson's Diversity Index (D), comparing healthy control (HC), SjD, and r‐axSpA patients per B cell population. The boxplots are presented with horizontal lines indicating the medians, with two hinges displaying the 25th and 75th percentiles, and jitter points reflecting study participants. The unpaired Wilcoxon test was used to compare the HC group with the SjD and r‐axSpA patient groups. *p*‐values <0.05 are considered significant. *<0.05, ***<0.001.

To investigate potential differences in clonal sizes between HCs and SjD or r‐axSpA patients, clones were divided into clonal groups based on the number of clonally‐related sequences (clonal members) across CD27^−^CD21^lo^ B cells, CD27^+^CD21^lo^ B cells, and PBs. The various B cell subsets were first normalized to the same number of unique sequences (i.e., clonally related sequences and singletons together), to take into account that there were differences in sequencing depth between the various subsets (see Methods: clonally related sequences and clonal relationships). Afterwards, clones were categorized as low clonality (2–5 clonal members), moderate clonality (6–20 clonal members), and high clonality (>20 clonal members). This analysis revealed that the number of clones with >20 members was significantly lower in SjD patients compared with HCs (Figure 1A). Subsequently, we evaluated the B cell subset distribution in the groups of clones with a low, moderate, or high clonality. The vast majority of clones with low clonality (2–5 members) represented CD27^−^CD21^lo^ B cells, whereas the majority of clones with high clonality represented PBs (Figure S2).

Next, we calculated the Shannon's and Simpson's diversity indexes, as proposed by Hill [25, 26, 27]. The Shannon's index is more sensitive to detect differences when B cell clones contain relatively few members, whereas Simpson's index is more sensitive to reveal whether clonal sizes are evenly distributed among B cell clones. Generally, a low index implies a lower diversity, as a consequence of more pronounced clonal expansion. When comparing CD27^−^CD21^lo^ B cells with CD27^+^CD21^lo^ B cells and PBs, we observed a relatively high diversity in CD27^−^CD21^lo^ B cells and much lower diversity in CD27^+^CD21^lo^ B cells and PBs based on the Shannon's and Simpson's indexes, as depicted for one representative SjD patient (Figure 1B,C). Compared with HCs, PBs from patients with SjD displayed a significantly higher Shannon (median 117.1, IQR 21.7–212.8 vs. median 378.7, IQR 262.5–494.9, respectively) and Simpson diversity index (median 59.7, IQR 38.8–80.6 vs. median 97.6, IQR 50.9–144.3, respectively; Figure 1C). This indicates that SjD patients contain more, but less expanded clones within the early‐stage PB subset compared with HCs.

Together, these results indicate that the CD27^−^CD21^lo^ B cell population contains smaller‐sized and distinct clones, leading to more repertoire diversity compared with CD27^+^CD21^lo^ B cells and PBs. No major differences were observed between HCs and patients with SjD or r‐axSpA, except for a small difference in clone size and diversity of PBs, and thus repertoire, between HCs and SjD patients.

### 2.2 CD27^−^CD21^lo^ B Cells Contain Largely Unmutated BCRs in HCs and Patients With SjD or R‐axSpA

**FIGURE 2:**
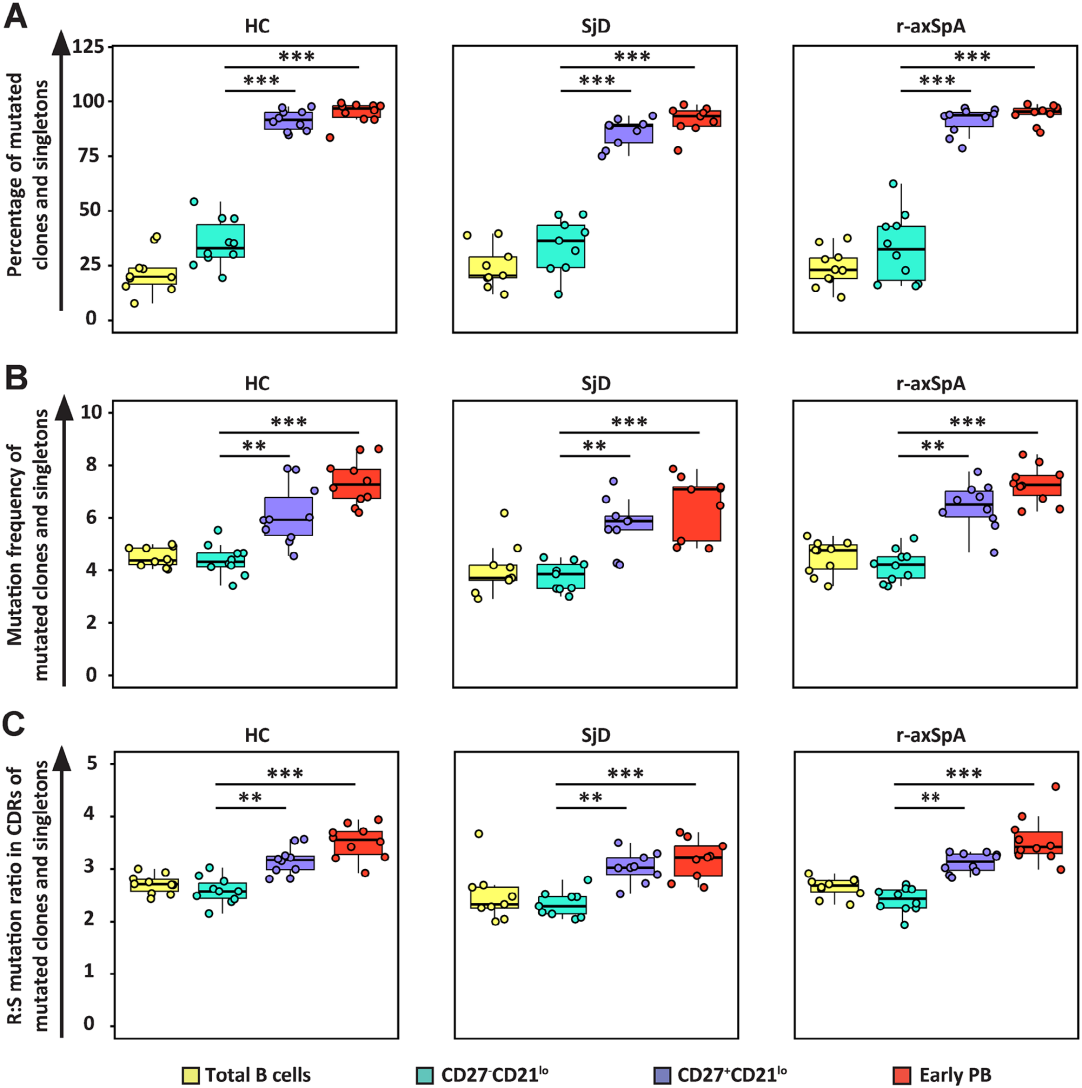
CD27^−^CD21^lo^ B cells are relatively less mutated compared with CD27^+^CD21^lo^ B cells and early‐stage PBs. (A) Boxplots demonstrating the percentages of unmutated (≤2 mutations) and mutated (>2 mutations) B cell clones and singletons derived from total B cells, CD27^−^CD21^lo^ B cells, CD27^+^CD21^lo^ B cells, and early PBs, shown for healthy controls (HC, *N* = 10), Sjögren's disease (SjD, *N* = 9), and radiographic axial spondyloarthritis (r‐axSpA), *N* = 10). (B) Boxplots depicting the average mutation frequency of mutated B cell clones and singletons from total B cells, CD27^−^CD21^lo^ B cells, CD27^+^CD21^lo^ B cells, and early PBs, for HCs, SjD, and r‐axSpA patients. (C) Boxplots depicting the average replacement‐to‐silent mutation ratio among B cell clones and singletons from total B cells, CD27^−^CD21^lo^ B cells, CD27^+^CD21^lo^ B cells, and early PBs, for HCs, SjD, and r‐axSpA patients. The boxplots are presented with horizontal lines indicating the medians, with two hinges displaying the 25th and 75th percentiles, and jitter points reflecting study participants. The Friedman test with Dunn's multiple comparisons correction was used to compare multiple B cell subsets within one study group. *p*‐values <0.05 are considered significant. **<0.01, ***<0.001.

We subsequently determined the mutational profile of V‐genes expressed by B cells from the various B cell subpopulations. For each set of clonally related sequences (clones), we took the mean number of mutations, to compensate for variation in clonality (number of clonal members). Total B cells were used as a reference group. Clones and singletons were classified as unmutated or mutated (≤2 or >2 mutations compared with their germline counterpart, respectively). As shown in Figure 2A, approximately one‐third of the sequences derived from CD27^−^CD21^lo^ B cells from HCs, SjD, and r‐axSpA patients were mutated. This is in marked contrast with CD27^+^CD21^lo^ B cells and early PBs, where on average 85% and 91%, respectively, of the sequences in the study groups were mutated. There were no statistical differences in the percentages of mutated clones and singletons between HCs and patients with SjD or r‐axSpA.

Next, we investigated whether the mutation frequency of the mutated (i.e., >2 mutations compared with their germline counterpart) singletons and mutated clonally related sequences (mean mutation number of the clone >2) was different among the three B cell populations. In HCs the mutated sequences of CD27^−^CD21^lo^ B cells harbored significantly fewer mutations compared with CD27^+^CD21^lo^ B cells and PBs (median mutation frequency of 4.3% (IQR 3.8–4.8), 5.9% (IQR 4.5–7.4), and 7.3% (IQR 6.2–8.4), respectively). A similar pattern was observed in patients with SjD or r‐axSpA patients (Figure 2B).

To see to what extent mutations in the B cell subpopulations were the result of an antigen‐driven process, we analyzed the replacement‐to‐silent mutations (R:S) ratio within the CDR regions (CDR1 and CDR2) of mutated (>2 mutations) singletons and clonally related sequences. In HCs, we observed that both CD27^+^CD21^lo^ B cells and PBs contained a significantly higher R:S ratio compared with CD27^−^CD21^lo^ B cells (Figure 2C). Similar patterns were found for patients with SjD or r‐axSpA patients, with no significant differences between the control and patient groups.

Taken together, these results indicate that the CD27^−^CD21^lo^ B cell population in HCs and patients with SjD or r‐axSpA consists of largely unmutated B cells, in contrast to CD27^+^CD21^lo^ B cells and PBs. The mutated fraction of CD27^−^CD21^lo^ B cells showed a significantly lower mutation frequency compared with mutated CD27^+^CD21^lo^ B cells and PBs. Also, antigen‐driven selection does not seem to vary between healthy individuals and patients in these three B cell subsets.

### 2.3 Biased IGHV and IGHJ Usage in Unmutated CD27^−^CD21^lo^ B Cell Sequences

Previous studies revealed a biased usage of the IGHV and IGHJ segments by BCRs in various autoimmune disorders [28]. Therefore, we analyzed the usage of IGHV and IGHJ genes in CD27^−^CD21^lo^ B cells, CD27^+^CD21^lo^ B cells, and early PBs, in the three groups of participants. For this analysis, all unique sequences (i.e. singletons and clonally related sequences) from a B cell subpopulation were used. As mentioned above, the repertoire of CD27^+^CD21^lo^ B cells and PBs was largely (>90%) composed of mutated singletons and clonally related sequences, whereas the repertoire of CD27^−^CD21^lo^ B cells comprised a significant proportion of unmutated sequences. Since clonal expansion and their associated mutations may be related to particular IGHV/IGHVJ genes, we analyzed the mutated and unmutated unique sequences (i.e., singletons and clonally related sequences) from CD27^−^CD21^lo^ B cells separately.

**FIGURE 3:**
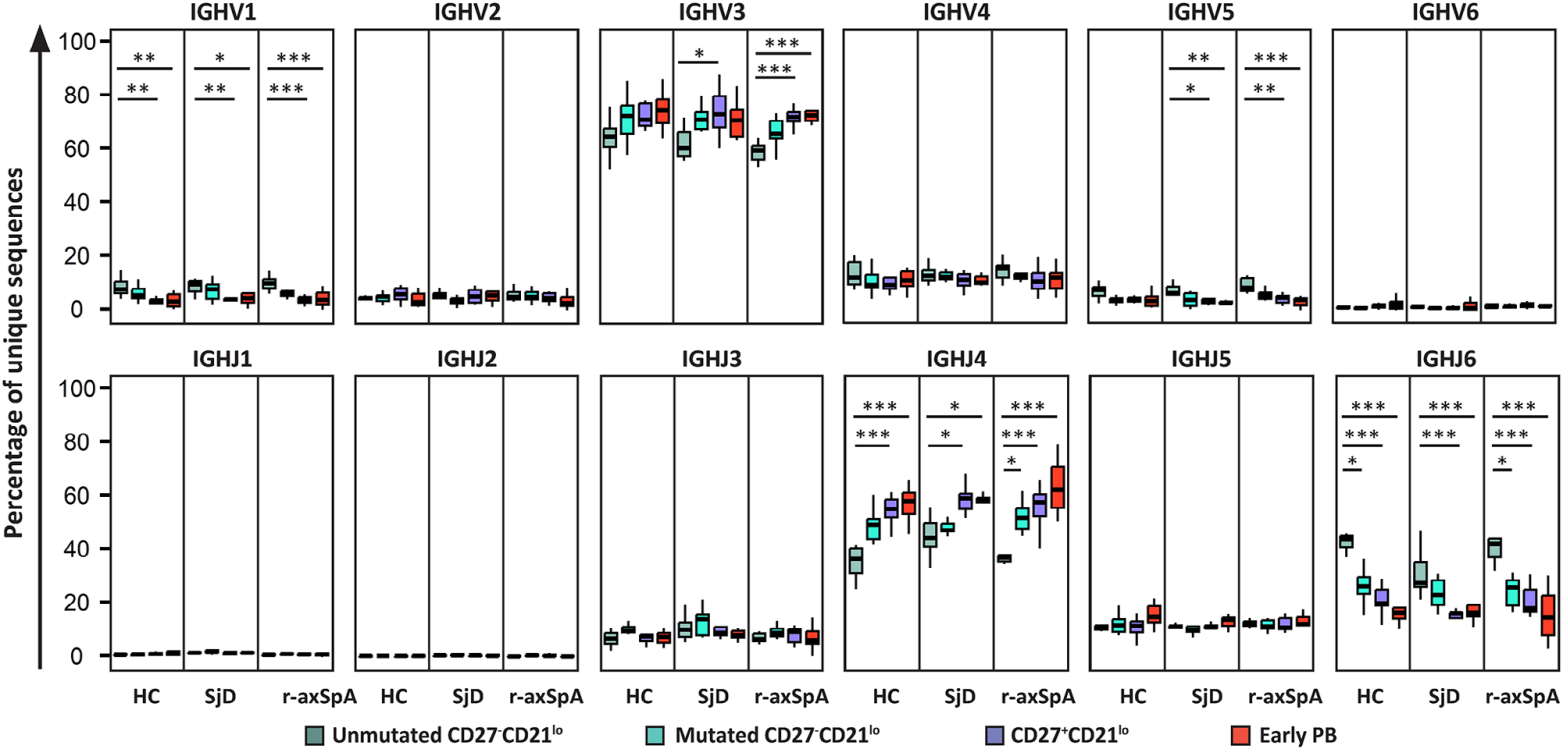
IGHV and IGHJ gene usage among unmutated and mutated CD27^−^CD21^lo^, CD27^+^CD21^lo^ B cells, and early‐stage PBs. Boxplots depicting the usage of IGHV (top plots) and IGHJ (bottom plots) segments by unique sequences (clonally related sequences and singletons, together) from unmutated and mutated CD27^−^CD21^lo^ B cells (≤2 mutations and >2 mutations compared with their germline, respectively), CD27^+^CD21^lo^ B cells and early PBs derived from healthy controls (HC, *n* = 10), Sjögren's disease (SjD, *n* = 9), and radiographic axial spondyloarthritis (r‐axSpA, *n* = 10) patients, as percentage of the total number of sequences. The boxplots are presented with horizontal lines indicating the medians, with two hinges displaying the 25th and 75th percentiles, and jitter points reflecting study participants. The Friedman test with Dunn's multiple comparisons correction was used to compare multiple B cell subsets within one study group. *p*‐values <0.05 are considered significant. *<0.05, **<0.01, ***<0.001.

In all B cell fractions that were analyzed (i.e., unmutated and mutated CD27^−^CD21^lo^ B cells, CD27^+^CD21^lo^ B cells, and PBs), most B cells used IGHV3 genes, accounting for approximately 60–65% of all unique sequences in HCs and patients with SjD or r‐axSpA (Figure 3). When comparing the various B cell fractions with each other (within the HCs or SjD or r‐axSpA patients), unmutated CD27^−^CD21^lo^ B cells used a significantly lower number of IGHV3 family genes and a higher number of IGHV1 and IGHV5 family genes compared with CD27^+^CD21^lo^ B cells and PBs (Figure 3). The percentages of IGHV‐family genes of the mutated CD27^−^CD21^lo^ B cells are mostly in‐between unmutated CD27^−^CD21^lo^ and CD27^+^CD21^lo^ B cells for every study group. No significant differences in IGHV‐family usage in the B cell fractions were seen between HCs and patients with SjD or r‐axSpA (Figure S3A). Nevertheless, an exploratory analysis of individual IGHV genes showed that the percentage of PBs that used the IGHV3‐64 gene was significantly elevated in the r‐axSpA patient group in comparison with HCs (median 3.0% [2.6–3.4 IQR] vs. median 0.7% [0.5–0.9 IQR]) (Figure 3B).

Subsequently, we determined the IGHJ family gene usage. BCR sequences from all analyzed B cell subpopulations showed the highest usage of IGHJ4 genes, in all groups of participants (Figure 3). There was a significantly lower usage of IGHJ4 genes and a higher usage of IGHJ6 genes in the fraction of unmutated CD27^−^CD21^lo^ B cells compared with other B cell fractions (Figure 3). Similar to the IGHV family gene analysis, the percentages of IGHJ genes of the mutated CD27^−^CD21^lo^ B cells are in‐between the unmutated CD27^−^CD21^lo^ and CD27^+^CD21^lo^ B cells. When comparing HCs with the two patient groups, we found that unmutated CD27^−^CD21^lo^ B cell sequences from SjD patients exhibited a significantly elevated usage of IGHJ4 genes (median 20.2% [19.6–20.8 IQR] in SjD vs. median 16.4% [16.2–16.6 IQR] in HC), which was accompanied by a significantly lower usage of IGHJ6 in this subpopulation (Supporting Information Figure S3A).

Taken together, our findings indicate that the unmutated CD27^−^CD21^lo^ B cell compartment has a different repertoire in terms of the IGHV family gene and IGHJ gene usage. Differences between the three groups of individuals were minimal for all B cell fractions, except for higher usage of IGHV3‐64 genes in r‐axSpA patients and differences in IGHJ gene usage between SjD patients and HCs.

### 2.4 Distinct CDR3 Length in CD27^−^CD21^lo^ B Cells from SjD Patients Compared with HCs

Given the importance of CDR3 in antigen recognition, investigating its length can provide insight into BCR structure that could contribute to or result from autoimmune pathology. To clarify whether CDR3 length differences exist between the CD21^lo^ B cell populations and PBs, as well as between healthy individuals and those with SjD or r‐axSpA, we analyzed the number of amino acids comprising the CDR3 region across each of these B cell populations (see Methods: Section 4.8). As argued in the previous paragraph, we analyzed the unique sequences obtained from unmutated (≤2 mutations) and mutated (>2 mutations) CD27^−^CD21^lo^ B cells separately. In HCs and r‐axSpA patients, CDR3 length was significantly shorter in mutated CD27^−^CD21^lo^ B cells compared with their unmutated counterparts (Supporting Information Figure S4). However, the CDR3 length of unmutated CD27^−^CD21^lo^ B cells from SjD patients was marked by a shorter number of amino acids than HCs and was similar to mutated cells. In general, even shorter lengths were observed in CD27^+^CD21^lo^ B cells compared with mutated CD27^−^CD21^lo^ B cells, although these differences were only significant for SjD and r‐axSpA patients. Likewise, PBs showed a short CDR3 length, which was comparable to CD27^+^CD21^lo^ B cells. There were no significant differences seen between HCs and r‐axSpA patients.

### 2.5 CD27^−^CD21^lo^ B Cells, CD27^+^CD21^lo^ B Cells, and Early Stage PBs Exhibit Clonal Relationships in HCs and Patients with SjD or R‐axSpA

To gain insight into the developmental relationships of CD27^−^CD21^lo^ B cells, CD27^+^CD21^lo^ B cells, and early‐stage PBs, we determined the presence of shared, clonally related sequences (clones) between these subsets. Of note, in this analysis also 100% identical (unmutated or mutated) sequences that may be present in two different B cell subpopulations (from one individual) indicate a clonal relationship. To account for differences in sequencing depth among the B cell subpopulations and individuals, we subsampled sequences to obtain a uniform sequence count (as described in the Methods: clonally related sequences and clonal relationships).

**FIGURE 4:**
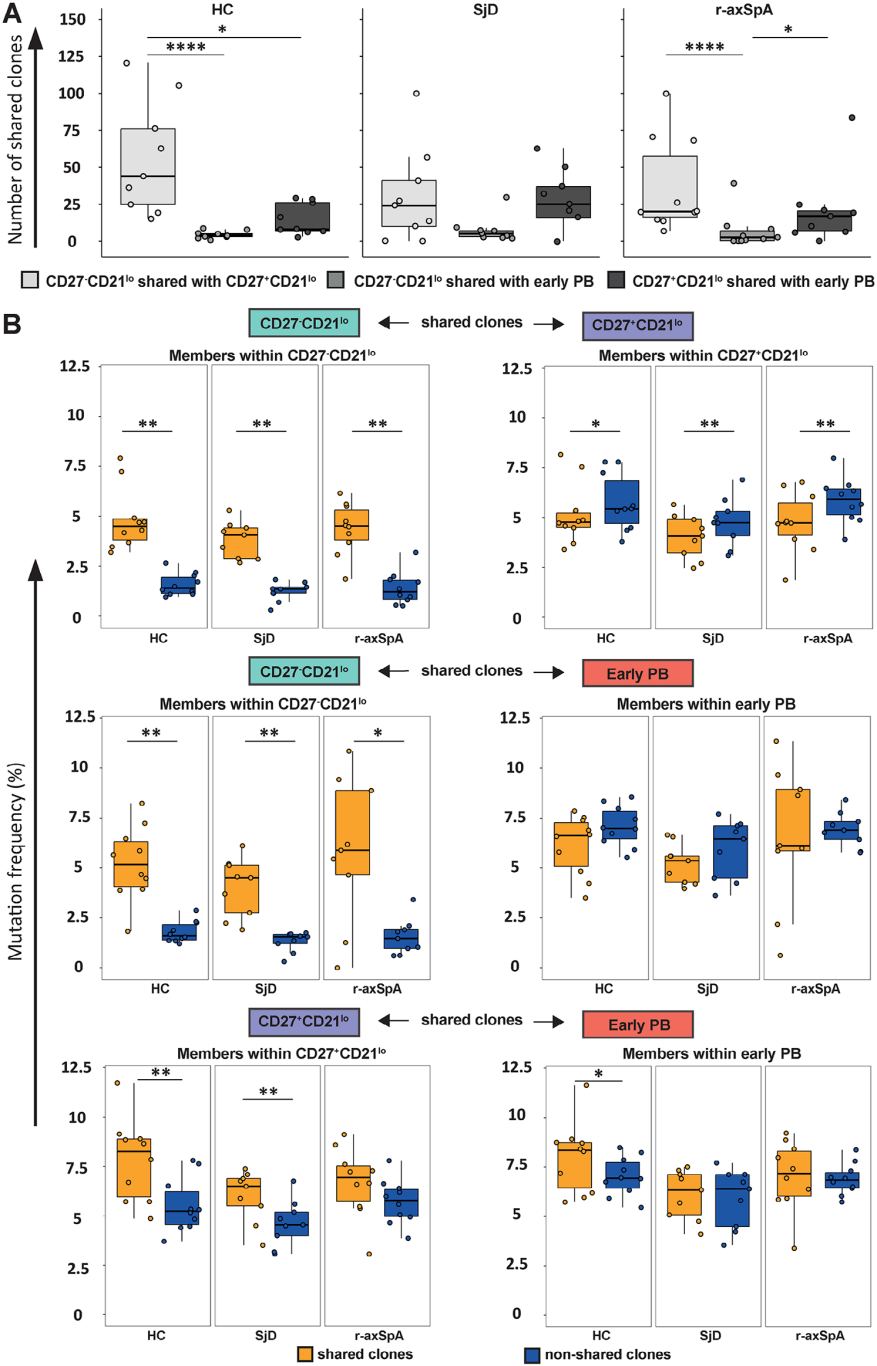
Clonal relationships are observed among CD27^−^CD21^lo^ B cells, CD27^+^CD21^lo^ B cells, and early‐stage PBs in HCs and patients with SjD or r‐axSpA. (A) Boxplots displaying the number of shared clones among CD27^−^CD21^lo^ B cells, CD27^+^CD21^lo^ B cells, and early (CD20^+^) PBs for healthy controls (*n* = 10), Sjögren's disease patients (*n* = 9), radiographic axial spondyloarthritis patients (r‐axSpA, *n* = 10). (B) Boxplots show the comparison of mutation frequencies between two groups, one containing the clones that share sequences with one or two other subsets (shared clones; orange), and the other comprising clones without shared sequences and including singletons (nonshared clones; blue). These comparisons are shown for the CD27^−^CD21^lo^ B cells, with shared clones related to either CD27^+^CD21^lo^ B cells and early PBs; the CD27^+^CD21^lo^ subset with shared clones related to CD27^−^CD21^lo^ B cells, and to early PBs; the early PB subset with shared clones related to CD27^−^CD21^lo^ B cells, and CD27^+^CD21^lo^ B cells. One r‐axSpA patient was omitted from the analyses between CD27^−^CD21^lo^ B cells and early PBs (the bottom two plots) because this study participant showed no shared clones between these two subsets. The boxplots are presented with horizontal lines indicating the medians, with two hinges displaying the 25th and 75th percentiles, and jitter points reflect the study participants. The Wilcoxon signed‐rank test was used to compare shared clones with nonoverlapping clones. *p*‐values <0.05 are considered significant. *<0.05, **<0.01, ****<0.0001.

To examine the degree to which CD27^−^CD21^lo^ B cells were related to CD27^+^CD21^lo^ B cells or PBs, we calculated the number of clones that were shared between these subpopulations (Figure S5). In HCs, the overall number of shared B cell clones was highest between CD27^−^CD21^lo^ B cells and CD27^+^CD21^lo^ B cells (median 44, IQR 25–76) (Figure 4A). Appreciable lower numbers of shared B cell clones were found between the CD27^−^CD21^lo^ B cell subpopulation and PBs (median 4, IQR 3–5) and, between the CD27^+^CD21^lo^ B cell subpopulation and PBs (median 8, IQR 7–26). In HCs, as well as SjD and r‐axSpA patients, the number of clones shared with PBs was higher for CD27^+^CD21^lo^ B cells compared with CD27^−^CD21^lo^ B cells (Figure 4A). No differences were observed in the number of shared clones among the subsets between the study groups. Of note, shared clones among the three B cell populations were observed in all SjD patients, and in the majority of HCs and patients with r‐axSpA (Supporting Information File S1, Sheet 6).

The presence of shared clones between CD27^−^CD21^lo^ B cells, CD27^+^CD21^lo^ B cells, and PBs led us to further investigate the mutational profile of these clones. Within a subpopulation, we compared the mutation frequency of sequences clonally related to another subpopulation (shared clones) with clonally related sequences that were not shared with that subpopulation (nonshared clones). For this analysis, we did not use the subsampled sequences but included clones derived from all unique sequences from the subpopulation in order to increase the reliability of the mutation frequency analysis. As shown in Figure 4B, in HCs, within the CD27^−^CD21^lo^ B cell subpopulation, the mutation frequency of shared clones (either with CD27^+^CD27^lo^ B cells or PBs) was significantly higher than nonshared clones. In contrast, within the CD27^+^CD21^lo^ B cell subpopulation of HCs, the shared clones exhibiting clonal relationships with CD27^−^CD21^lo^ B cells showed a significantly lower mutation frequency than nonshared clones. Interestingly, within the CD27^+^CD21^lo^ B cell subpopulation of HCs, clones shared with PBs had a significantly higher mutation frequency than nonshared clones. These differences in mutation frequencies were also observed in patients with SjD or r‐axSpA.

We hypothesized that the CD27^−^CD21^lo^ B cell population is a precursor pool to CD27^+^CD21^lo^ B cells and/or PBs. To investigate this, we generated phylogenetic clonal lineage trees of clones categorized with a high clonality (comprising >20 clonally‐related sequences) and when they included at least one clonally‐related sequence from each of the three B cell populations. The clonal lineage trees generally showed that CD27^–^CD21^lo^ B cells are closest to the germline sequence, while CD27^+^CD21^lo^ B cells and PBs are progressively more mutated, supporting the hypothesis that CD27^–^CD21^lo^ B cells may transition into CD27^+^CD21^lo^ B cells and/or PBs (as shown in Figure S7, one tree from study participants of each respective study group). However, we also observed some instances where PBs were not the most mutated members within a clone, as seen for a clone from one healthy individual (Figure S6A).

Collectively, these data show that clones are shared between the investigated B cell subpopulations, and that CD27^–^CD21^lo^ B cells possibly transition into CD27^+^CD21^lo^ B cells and/or PBs, which are typically associated with increased mutation frequency.

## 3 Discussion

Increased frequencies of circulating CD21^lo^ B cells are observed in multiple rheumatic diseases, including SjD and r‐axSpA. The CD21^lo^ B cell population exhibits heterogeneity and comprises both CD27^−^ and CD27^+^ cells. Although a lack of CD27 expression is usually associated with naïve B cells, some memory B cell subsets may also have a CD27^−^ phenotype [13, 29]. The aim of this study was to gain insight into the origin of CD27^−^CD21^lo^ B cells and their connection with CD27^+^CD21^lo^ B cells and early‐stage PBs. By performing BCR repertoire analysis, we showed that the CD27^−^CD21^lo^ B cell subpopulation comprised more smaller‐sized clones and singletons compared with CD27^+^CD21^lo^ B cells and PBs, both in healthy and diseased individuals. Furthermore, whereas roughly one‐third of the clones and singletons of the CD27^−^CD21^lo^ B cells carried mutated IGHV sequences, >85% of the sequences derived from CD27^+^CD21^lo^ B cells and PBs were mutated, in all three study groups (HC, SjD, and r‐axSpA). In addition, in the mutated sequences of CD27^−^CD21^lo^ B cells, the mutation frequency was lower than in CD27^+^CD21^lo^ B cells and PBs. Together, these data suggest that the CD27^−^CD21^lo^ B cell subpopulation contains a large proportion of unmutated cells, in contrast to CD27^+^CD21^lo^ B cells and PBs. Finally, in both healthy individuals and patients, we were able to demonstrate clonal relationships between cells in all three subsets; CD27^−^CD21^lo^ B cells, CD27^+^CD21^lo^ B cells, and PBs. In HCs, most shared clones were observed between CD27^−^CD21^lo^ B cells and CD27^+^CD21^lo^ B cells, and we found a significantly lower number of shared clones between CD27^−^CD21^lo^ B cells and PBs and between CD27^+^CD21^lo^ B cells and PBs. Furthermore, our results indicate that the relation with PBs seems more evident in CD27^+^CD21^lo^ B cells, reflected by a trend toward elevated numbers of shared clones in SjD and r‐axSpA patients, as well as HCs, compared with CD27^−^CD21^lo^ B cells. These findings suggest that both CD27^−^CD21^lo^ and CD27^+^CD21^lo^ B cells may develop into PBs and, therefore, actively contribute to immune responses in patients and healthy individuals.

We observed that the CDR3 length of unmutated CD27⁻CD21^lo^ B cells from SjD patients was shorter compared with HCs, and comparable to that of mutated CD27⁻CD21^lo^ B cells in the same patients. In SjD patients, the CDR3 length of unmutated CD27⁻CD21^lo^ B cells aligned with the previously reported length of naïve B cells [10]. Notably, Ota et al. [10] demonstrated that naïve B cells from SjD patients have a significantly shorter CDR3 region compared with those cells from HCs. Our findings corroborate this, showing that unmutated CD27⁻CD21^lo^ B cells from SjD patients also exhibit significantly shorter CDR3 lengths than HCs. This suggests that the unmutated CD27⁻CD21^lo^ B cell population includes naïve B cells, and further supports the idea that the reduction in CDR3 length seen in SjD patients may be a common feature across unmutated B cells. Ota et al. [10] proposed that the shorter CDR3 length in naïve B cells of SjD patients may result from effects of elevated interferon activity in the bone marrow. We did not observe this shortening in r‐axSpA patients, indicating that these patients are not similarly affected.

As expected, and shown here, the majority (>85%) of CD27^+^CD21^lo^ B cells express mutated BCRs. The isotype of the heavy chain of these cells could not be established with the approach that we used, but previously we demonstrated that roughly half of these cells are isotype‐switched [18]. Thus, CD27^+^CD21^lo^ B cells are comprised of both bona fide isotype‐switched memory cells and unswitched memory cells. Typically, resting memory B cells express high levels of CD21 [30]. Low expression of CD21 on memory B cells may be the result of (recent) activation [31]. CD27^+^CD21^lo^ B cells may be derived from germinal centers and primed to develop toward plasma cells [17]. The high number of clones containing a high number (>20) of clonally‐related sequences within the CD27^+^CD21^lo^ B cells subpopulation suggests that some of these cells have undergone clonal selection and expansion in all study groups. Also, we showed that B cell clones can have members in both the CD27^+^CD21^lo^ and PB cell fractions (shared clones). Furthermore, shared clones between CD27^+^CD21^lo^ cells and PBs had a higher number of mutations compared with nonshared clones in the CD27^+^CD21^lo^ B cell population. These findings provide evidence for the differentiation of highly mutated CD27^+^CD21^lo^ cells toward PBs, suggestive of involvement in active immune responses.

In marked contrast to the CD27^+^CD21^lo^ subpopulation, significantly fewer B cells in the CD27^−^CD21^lo^ fraction were mutated in all study groups, and approximately half of the BCR sequences were in their germline configuration. The remaining sequences carried relatively few mutations. The presence of mutated CD27^−^CD21^lo^ B cells in both control and patient groups is consistent with findings by Sutton et al. [32], demonstrating that the somatic mutation frequency in this population of cells was significantly higher compared with naïve B cells, as observed in HCs. Also, our previous flow cytometry analysis demonstrated that a large proportion (∼80%) of the CD27^−^CD21^lo^ B cells in HCs, SjD, and axSpA patients are unswitched and express IgM and/or IgD [18]. In the current study, it is plausible that the unswitched, unmutated cells within this fraction are either (activated) naïve cells or possibly anergic cells, negatively selected because of autoreactivity. Several studies demonstrated that part of the CD27^−^CD21^lo^ B cells exhibits autoreactive BCRs and anergic characteristics, as marked by a lack of calcium efflux and diminished upregulation of B cell activation markers and proliferation upon stimulation of the BCR [14, 16]. However, CD27^−^CD21^lo^ B cells may escape anergy when BCR stimulation is combined with other stimuli, for example, TLR agonists and cytokines [14, 16, 33].

The mutated CD27^−^CD21^lo^ B cells are apparently the result of an immune response during which these cells acquired mutations in the BCR. At least part, if not all, of the mutated cells may well represent double‐negative 2 (DN2) B cells, which are expanded in the circulation of patients with autoimmune diseases such as SLE and display mutated BCRs [33]. These cells are defined as DN (CD27^−^IgD^−^) B cells and are further characterized by CD11c expression and a lack of CD21 and CXCR5 expression. Indeed, we found previously that the isotype‐switched (IgD^−^IgM^−^) fraction of CD27^−^CD21^lo^ B cells is marked by the expression of CD11c (and T‐bet) and a lower expression of CXCR5 [18]. Furthermore, DN2 B cells have been shown to respond to BCR stimulation in the presence of TLR7 agonists and cytokines such as IFNγ and IL‐21.

A study by Jenks et al. reported a substantial degree of clonal connectivity between DN2 cells and plasma cells in particular in patients with SLE, and they speculated that these cells were, in fact, preplasmablasts [33]. In our study, we could confirm a direct relationship between CD27^−^CD21^lo^ B cells and PBs in healthy individuals and in patients with SjD or r‐axSpA. However, the numbers of shared clones between CD27^−^CD21^lo^ B cells and PBs were significantly lower compared with CD27^−^CD21^lo^ B cells and CD27^+^CD21^lo^ B cells, not only in HCs but also in patients r‐axSpA. Although SjD patients showed a similar pattern, the difference in numbers did not reach significance. Within the CD27^−^CD21^lo^ fraction, mutation rates of shared clones were higher compared with nonshared clones of this fraction, but still lower compared with nonshared clones of the CD27^+^CD21^lo^ fraction. These findings may argue for a preferential differentiation of the (mutated) CD27^−^CD21^lo^ B cells toward CD27^+^CD21^lo^ B cells, rather than a direct differentiation toward PBs. It also argues against differentiation the other way around, from CD27^+^CD21^lo^ B cells toward CD27^−^CD21^lo^ B cells. Furthermore, the number of shared clones with PBs was higher for CD27^+^CD21^lo^ B cells than for CD27^−^CD21^lo^ B cells, supporting the notion of CD27^+^CD21^lo^ B cells to PBs as the preferred route. Clonal lineage analysis showed that sequences from CD27^−^CD21^lo^ B cells were generally closer to the clonal germline compared with those from CD27^+^CD21^lo^ B cells and PBs. This suggests that the CD27^−^CD21^lo^ B cell population serves as a reservoir of precursor cells for these more mature populations. Because CD27^−^CD21^lo^ B cells are expanded in patients with SjD and r‐axSpA, this may lead to an increased precursor pool for CD27^+^CD21^lo^ B cells and PBs in absolute terms, within the context of immune‐mediated disease.

The relatively low mutation rate among mutated CD27^−^CD21^lo^ B cells may be the result of germinal center‐independent extrafollicular B cell responses or early exit of cells out of the germinal center. Experimental studies in mice indicated that the introduction of somatic mutation in BCRs is not exclusive to germinal centers, but may also occur during extrafollicular responses [34, 35, 36]. DN2 B cells are associated with an extrafollicular response and are reported to demonstrate a lower frequency of somatic mutations compared with classical switched memory B cells [33]. This aligns with our finding of lower mutation frequencies in the mutated CD27^−^CD21^lo^ B cells compared with CD27^+^CD21^lo^ B cells. However, in contrast to DN2 B cells from SLE patients, which seem to have a mutation frequency similar to plasma cells, we observed a much lower mutation frequency in mutated CD27^−^CD21^lo^ B cells compared with PBs in HCs, and SjD or r‐axSpA patients [33].

Our study is subject to limitations. We recognize that due to our sequencing approach, we were unable to determine the constant regions of the BCRs, which prevented analysis of the isotype distribution. Also, we restricted our investigation to CD27^−^CD21^lo^ B cells, CD27^+^CD21^lo^ B cells, and PBs to obtain insight into the BCR repertoire of and relationships among these B cell populations. The sorting strategy employed included CD19^+^CD20^+^ B cells. With this approach, we focused on relatively immature PBs that were recently generated. However, since mature PBs are largely CD20^lo/−^, this approach may have limited the resolution of PB clonality. A more intricate sorting strategy or single‐cell multi‐omics approach might provide a better understanding of the nature and origin of CD27^−^CD21^lo^ B cells. Moreover, whilst the number of controls and patients included in this study was relatively high compared with other studies investigating BCR repertoires, the number of patients was insufficient to study clinical subtypes.

In conclusion, the results of this study suggest that in healthy individuals, as well as in SjD and r‐axSpA patients, CD27^−^CD21^lo^ B cells are clonally related to CD27^+^CD21^lo^ B cells and early‐stage PBs. Repertoire diversity analysis and phylogenetic trees indicate a developmental progression from CD27^−^CD21^lo^ B cells toward CD27^+^CD21^lo^ B cells and/or to PBs. Given the known expansion of CD27^−^CD21^lo^ B cells in the peripheral blood of SjD and r‐axSpA patients, their association with autoreactivity and their potential to differentiate into CD27^+^CD21^lo^ B cells and PBs, suggests that these cells may actively contribute to immune responses. Future research should focus on the specific contribution of CD27^−^CD21^lo^ B cells to SjD and r‐axSpA pathogenesis. In this regard, we encourage the investigation of CD21^lo^ B cells at inflammatory sites predisposed to SjD or r‐axSpA.

## 4 Material and Methods

### 4.1 Patients and Healthy Control Samples

Buffy coats were obtained from 10 HCs via the Sanquin Blood Supply Foundation in the Netherlands. In addition, we collected whole blood from 10 SjD patients included in the Registry of Sjögren's Disease in UMCG LongiTudinal cohort, who fulfilled the 2016 ACR‐EULAR classification criteria [37]. Furthermore, we included 10 r‐axSpA patients enrolled in the Groningen‐Leeuwarden Axial Spondyloarthritis cohort who fulfilled the ASAS classification criteria for axial spondyloarthritis [38]. Patients with SjD or r‐axSpA were selected on the basis of exhibiting varying levels of disease activity (based on EULAR Sjögren's Syndrome Disease Activity Index [ESSDAI] or Ankylosing Spondylitis Disease Activity Score [ASDAS], respectively). Neither SjD nor r‐axSpA patients received any biological disease‐modifying anti‐rheumatic drug (DMARDs) or other immunosuppressant, such as prednisolone, within six months preceding sampling. PBMCs were isolated from either buffy coats (for HCs) or whole blood and were subsequently cryopreserved at −150°C until further use. The medical research ethics committee of the Medical Center Leeuwarden provided approval for the study of HCs and r‐axSpA patients (RTPO 364/604), and the University Medical Center Groningen for studying patients with SjD (METc 2014.491). All HCs and patient participants provided written informed consent in accordance with the Declaration of Helsinki. Patient characteristics are summarized in Table 1. Details of individual study participants are provided in Supporting Information File S1, Sheets 1–3.

**TABLE 1:** Clinical characteristics of healthy controls and patient groups.

Characteristics	HC	SjD	r‐axSpA
Number of participants (*n*)	10	10	10
Age in years, mean ± SD	48 ± 21	51 ± 12	46 ± 9
Male gender (*n*)	6	0	6
Symptom duration in years, median (IQR)		11.4 (6.2–15.9)	22.0 (13.3–25.8)
ESR (mm/h), median (IQR)		40.5 (36.5–71.3)	13.0 (7.0–17.8)
CRP (mg/L), median (IQR)		3.0 (1.3–14.8)	2.0 (1.0–4.3)
Naïve for biological DMARDs (*n*)		7	5
SjD specific features:			
ESSDAI, median (IQR)		6.5 (2.3–10.0)	
Anti‐SSA positive (*n*)		10	
Anti‐SSB positive (*n*)		6	
RF positive (>5 IU/mL) (*n*)		9	
IgG (g/L), mean ± SD		19.5 ± 5.5	
r‐axSpA specific features:			
HLA‐B27 positive (*n*)			8
ASDAS, mean ± SD			2.2 ± 0.8
BASDAI, mean ± SD			4.7 ± 2.5
History of:			
Uveitis (*n*)			4
Inflammatory bowel disease (*n*)			1
Psoriasis (*n*)			0
≥1 ESM (*n*)			5

*Note*: Data are presented as number of participants (*n*), mean ± SD, or median (IQR).

Abbreviations: anti‐SSA/Ro, anti‐Sjögren's‐syndrome‐related antigen A autoantibodies; anti‐SSB/La, anti‐Sjögren's‐syndrome‐related antigen B autoantibodies; ASDAS, Ankylosing Spondylitis Disease Activity Score; BASDAI, Bath Ankylosing Spondylitis Disease Activity Index; DMARD, disease‐modifying antirheumatic drugs; ESM, extraskeletal manifestations; ESSDAI; EULAR Sjögren's syndrome disease activity index; HC, healthy controls; HLA‐B27, human leukocyte antigen B27; N/A, not assessed; r‐axSpA, radiographic axial spondyloarthritis; RF, rheumatoid factor; SjD, Sjögren's disease.

### 4.2 Isolation and Sorting of B Cell Populations

Cryopreserved PBMCs were thawed, followed by B cell enrichment through T cell depletion, using the EasySep™ human CD3 immunomagnetic positive selection kit (StemCell Technologies, Germany). The B cell‐enriched cell fraction was stained for surface markers, described in Table S2. Subsequently, different B cell populations were sorted utilizing the MoFlo Astrios cell sorter (Beckman Coulter), namely total CD19^+^CD20^+^ B cells, CD27^−^CD38^lo^CD21^lo^ B cells, CD27^+^CD38^lo^CD21^lo^ B cells, and early stage PBs gated as CD27^+^CD38^++^. For the CD21^lo^ populations, we selected CD38^lo^ cells to exclude transitional B cells (CD38^++^). CD27^−^CD38^lo^CD21^lo^ B cells will be further referred to as CD27^−^CD21^lo^ B cells and CD27^+^CD38^lo^CD21^lo^ B cells as CD27^+^CD21^lo^ B cells. The gating strategy for sorting is depicted in Figure S1, and gates were set based on fluorescence‐minus‐one controls. The sorted B cell populations were captured in safe‐lock Eppendorf tubes (Qiagen, the Netherlands) containing 350 uL RNeasy Lysis Buffer (RLT, Qiagen). Immediately postsorting, β‐mercaptoethanol (10 uL/mL) was added to denature RNAses. Lysates were stored at −80°C for preservation.

### 4.3 RNA Isolation and Next‐Generation Sequencing

To increase the yield of the RNA extraction procedure of the sorted B cell fractions, 100,000 non‐BCR expressing HEK293T cells were added to each sample prior to RNA isolation (RNeasy Micro Kit, Qiagen). RNA sequencing of the BCR heavy chains was performed to obtain unique molecular identifier (UMIs)‐tagged products [39]. Briefly, specific complementary DNA (specific‐cDNA) of BCR sequences was synthesized using a BCR heavy chain joining gene reverse primer tagged with a UMI (nine random nucleotides) and a consensus sequence. After specific cDNA synthesis, Exonuclease I (Thermo Fisher Scientific) treatment was performed to remove the remaining primers. This step was followed by a multiplexed PCR with six forward primers covering all BCR heavy chain variable genes and a reverse primer binding to the same primer as used for specific‐cDNA synthesis and tagged with an eight base pair patient identifier (MID, Molecular Identifier; details on the primers available upon request from the corresponding author). Obtained amplicons were purified using two rounds of AMPure XP beads clean‐up (Beckman Coulter), quantified using Qubit dsDNA HS Assay Kit (Thermo Fisher Scientific), dual‐indexed with i5 and i7 adapters (Nextera XT Index Kit v2) and sequenced using the Illumina Miseq Kit v3 2 × 300 bp technology according to the manufacturer's manual.

### 4.4 Bioinformatic Processing of BCR Sequences

Preprocessing of raw sequences was conducted using pRESTO (v0.7.0) [40]. The primary workflow comprised several steps including quality control (removing reads with a mean Phred quality score <20), primer masking, sequence annotation, and the generation of consensus sequences that are annotated with identical UMIs. For each sorted B cell subset per study participant, paired‐end reads were assembled, sequences with over 20 missing nucleotides were discarded, and read counts for each group of sequences with identical UMIs were aggregated. Subsequently, duplicate UMI‐collapsed sequences were removed. The remaining sequences were considered unique sequences. VDJ germline genes were assigned to reconstruct VDJ sequences using IgBLAST (v1.19.0), using the December 1, 2019 version of the IMGT gene database. Nonproductive sequences were excluded from subsequent analysis. The CreateGermlines.py function within Change‐O (v1.3.0) was used to reconstruct germline sequences for each V(D)J sequence, with D segment and N/P regions masked (using Ns) [41]. A contamination assessment was conducted for unrelated samples. The final unique sequence counts that passed the quality control and filtering steps are found in Supporting Information File S1, Sheet 4.

### 4.5 Clonally Related Sequences and Clonal Relationships

We used the Change‐O toolkit to identify the presence of clonally related sequences among the unique sequences [41]. The immunoglobulin heavy chains are sufficient to determine clonal relationships [42]. For each study participant, the unique sequences from the different subsets were compiled into a single dataset. Subsequently, unique sequences were grouped based on shared V and J gene annotations and identical junction lengths. Within these groups, unique sequences were clustered as clonally related sequences (derived from clonally related cells, forming clones) if the difference between sequences fell within a single‐nucleotide normalized Hamming distance threshold. This threshold was based on the number of mismatched sequences and reconstructed germline sequences (described in the section above). Furthermore, the Hamming distance threshold was determined for each participant by identifying the minima situated between the two modes within the bimodal distance‐to‐nearest histogram. This minimum reflects the optimal Hamming distance threshold to separate clonally related sequences from other unique sequences. Afterward, clones were inferred through the Change‐O's DefineClone.py function that performed hierarchical clustering with single‐linkage, using the optimal Hamming distance threshold, specifically determined for each individual. In this study, we defined “clones” as groups of clonally related sequences, and the remaining unique sequences are called “singletons”. When clones consist of clonally related sequences from different B cell subsets, we term these “overlapping clones”. Conversely, clones that comprise only clonally related sequences from a single subset are designated as “nonoverlapping clones”.

To investigate clonal relationships among B cells from different subsets (within one individual), we quantified the number of shared clonally related sequences among CD27^−^CD21^lo^ B cells, CD27^+^CD21^lo^ B cells, and PBs. Due to potential differences in sequencing depth among the samples, we subsampled the unique sequences randomly to a uniform arbitrary count of 3195 sequences per B cell subset from each study participant prior to data analysis. Subsequently, clonally related sequences were identified using Change‐O's toolkit [41], as described above. Afterward, the number of shared clones was calculated amongst the three B cell subsets per study group.

To investigate clonal lineages, we reconstructed phylogenetic clonal lineage trees using Dowser, part of the Immcantation pipeline [43]. For the reconstruction of trees, all unique sequences per B cell population (except for the total B cell population) of each study participant were used. Furthermore, trees were built using IgPhyML, which incorporates somatic hypermutation hotspot and coldspot motifs [44].

### 4.6 Clonal Size and Diversity Analysis

For the clonal size and diversity analyses, we accounted for differences in sequencing depth, unique sequences were subsampled to a uniform count of 3195 unique sequences per B cell subset within a study participant. The samples that did not meet the subsampled threshold are reported in Supporting Information File S1, Sheet 5. The repertoire diversity analysis of unique sequences within a B cell subset was performed using the Alakazam R‐package [41]. To this end, the unique sequences from different individuals were analyzed per subset. Initially, for each subset, the clonal abundance was determined by calculating the number of clonally related sequences of each clone, divided by the total number of unique sequences. Subsequently, abundance distributions were derived from the fractions of each clone and singleton per study participant, including 95% confidence intervals, through bootstrapping (1000 iterations). Afterward, both Shannon's and Simpson's (reciprocal) diversity indexes as proposed by Hill were computed, which are derived from the clonal abundance distributions of healthy individuals and patients [27]. These two indexes were calculated for each B cell subset from each study participant [27]. Shannon's and Simpon's metrics are both measurements of diversity in an immune repertoire, with a lower index indicative of less diversity and elevated clonal expansion [25, 26]. For each participant, both Shannon's and Simpon's index distributions were based on the average value of 1000 resampling iterations, with a 95% confidence interval calculated using the Z‐score, derived from the variance of the bootstrap distribution.

### 4.7 Mutation Profile Analysis

The number and frequency of mutations in BCR sequences were calculated as the number of mismatches per base pair within the V segment compared with the germline sequence, excluding the N/P and D regions, using the SHazaM R‐package [41]. We chose to calculate mutations for the V segment (up to the start of the junction), instead of the V(D)J sequence, because the inferred germline state of the junction is less reliable [26]. To analyze mutation frequencies for each B cell subset per individual, we used the set of sequences that were used for the abundance and diversity analysis. We first calculated for each clone within a B cell subset the mean mutation frequency of all sequences forming that clone. Subsequently, the mean mutation frequency of each clone was used as a unique frequency. We adopted this approach to avoid skewing of the results by larger‐sized clones. Next, to obtain the mutation frequency per cell subset per individual, we averaged the mean mutation frequencies of all B cell clones and remaining unique sequences. Furthermore, to investigate whether antigen‐driven selection plays a role in this process, we analyzed the replacement‐to‐silent mutations by dividing the replacement mutations by the silent mutations within the CDR regions (CDR1 and CDR2).

### 4.8 V and J Gene Usage and Heavy Chain CDR3 Length Analysis

We used the Alakazam R‐package to analyze the V and J gene usage and the heavy chain CDR3 length of unique sequences from the different B cell subsets per individual [41]. The V and J gene families and individual gene usage were calculated as a percentage of the unique sequences per B cell population for each given study participant. To perform the CDR3 sequence length analysis, we reduced the junction sequence to the CDR3 sequence by removing the first and last codon (the conserved residues) using the trim = TRUE argument within the aminoAcidProperties function.

### 4.9 Statistical Analysis

Data analysis and statistical tests were conducted using R software (version 4.1.1). Data are presented as mean ± standard deviation (SD) or median with interquartile range (IQR), depending on the distribution characteristics of variables. When comparing two independent groups, we applied the Mann–Whitney *U* test, and for dependent (related) groups we used the Wilcoxon signed‐rank test. For comparisons involving more than two independent groups, a Kruskal–Wallis test with Dunn's adjustment for multiple testing was used. When comparing three or more (related) groups, such as three B‐cell subsets within one individual, a Friedman test with Dunn's multiple comparisons test was performed. The statistical test(s) used for the analyses are described in the Figure captions. *p*‐values less than 0.05 were considered statistically significant.

## Author Contributions

Conceptualization: Rick Wilbrink, Anneke J. P. L. Spoorenberg, Gwenny M. P. J. Verstappen, and Frans G. M. Kroese. Experiments: Rick Wilbrink and Ilse T. G. Niewold. Data analysis: Rick Wilbrink and Linda van der Weele. Writing original draft: Rick Wilbrink. Review and editing: Linda van der Weele, Gwenny M. P. J. Verstappen, Frans G. M. Kroese, Anneke J. P. L. Spoorenberg, and Niek de Vries. All authors have read and agreed to the published version of the manuscript.

## Conflicts of Interest

A.S. has received grant/research support from Abbvie, Pfizer, Union Chimique Belge (UCB), and Novartis and acted as a consultant for Abbvie, Pfizer, MSD, UCB, Lilly, and Novartis. F.K. has received unrestricted grants from BMS, is a consultant for BMS, speaker for BMS Roche and Jannsen‐Cilag. The remaining authors declare no conflicts of interest.

## Ethics Approval Statement

The medical research ethics committee of the Medical Center Leeuwarden provided approval for the study of HCs and r‐axSpA patients (RTPO 364/604), and the University Medical Center Groningen for studying patients with SjD (METc 2014.491).

## Patient Consent Statement

All HCs and patient participants provided written informed consent in accordance with the Declaration of Helsinki.

### Peer Review

The peer review history for this article is available at https://publons.com/publon/10.1002/eji.202451398.

## Supporting information



Supporting Information

Supporting Information

## Data Availability

The data that support the findings of this study are available from the corresponding author upon reasonable request.
