# Supplementary material for: B Cell Receptor Repertoire Analysis of the CD21lo B Cell Compartment in Healthy Individuals, Patients With Sjögren's Disease, and Patients With Radiographic Axial Spondyloarthritis
Source: Eur J Immunol. 2024 Dec 20;55(2):e202451398. doi: 10.1002/eji.202451398 (PMC11830390; doi:10.1002/eji.202451398)
Supplement: Supplementary file 2 — Supporting Information [file EJI-55-e202451398-s002.pdf]

Supplementary materials to:

B cell receptor repertoire analysis of the CD21^{lo} B cell compartment in healthy individuals, patients with Sjögren's disease and patients with radiographic axial spondyloarthritis

Rick Wilbrink[1], Linda van der Weele[2], Anneke J.P.L. Spoorenberg[1], Niek de Vries[2], Ilse T.G. Niewold[2], Gwenny M.P.J. Verstappen[1*] and Frans G.M. Kroese[1*]

*Co-senior author

[1]Department of Rheumatology and Clinical Immunology, University of Groningen, University Medical Center Groningen, Groningen, The Netherlands

[2] Department of Rheumatology & Clinical Immunology, Amsterdam Rheumatology and Immunology Center (ARC), Amsterdam UMC, University of Amsterdam, Amsterdam, The Netherlands

Keywords: B cells, BCR repertoire, CD21^{lo} B cells, Sjögren's disease, radiographic axial spondyloarthritis

Supplementary Tables:

Supplementary Table 1 | Mean percentage of clonally related sequences among all unique sequences calculated per B cell subset and per study group.

**Group**	**Total B cells**	**$CD27^{-}CD21^{lo}$**	**$CD27^{+}CD21^{lo}$**	**Early PB**
HC (n=10)	48 ± 9	58 ± 18	85 ± 5	96 ± 1
SjD (n=9)	54 ± 9	52 ± 13	83 ± 9	93 ± 5
r-axSpA (n=10)	53 ± 7	57 ± 14	84 ± 10	96 ± 2

Data are presented as mean ± SD; HC = healthy controls; SjD = Sjögren’s Disease; r-axSpA = radiographic axial spondyloarthritis.

Supplementary Table 2 | Antibodies used for sorting B cell populations

**Immune marker**	**Fluorochrome**	**Clone**	**Company**	**Catalog#**
CD19	FITCH	HIB19	Biolegend	302206
CD20	PE	2H7	eBioscience	12-0209-73
CD21	BV421	B-ly4	BD Horizon	562966
CD27	APC	O323	Biolegend	302810
CD38	PE/Cyanine7	HIT2	Biolegend	303516

Antibodies were stored and handled according to the product information.

Supplementary Figures:

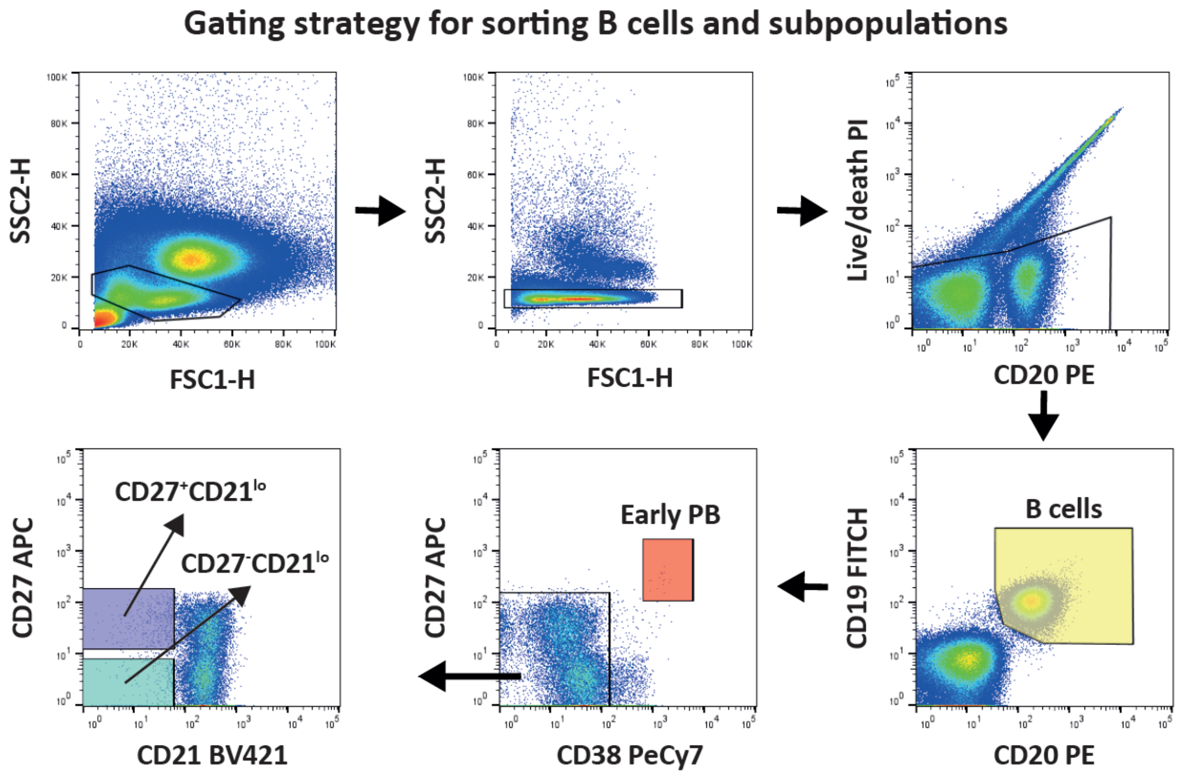

Supplementary Figure 1. Gating Strategy for Sorting B Cells and Subpopulations. The sorted populations include B cells (yellow), early plasmablasts (PB; red), CD27^{+}CD21^{lo} B cells (purple), and CD27^{-}CD21^{lo} B cells (turquoise). Arrows indicate the progression to the next level of gating.

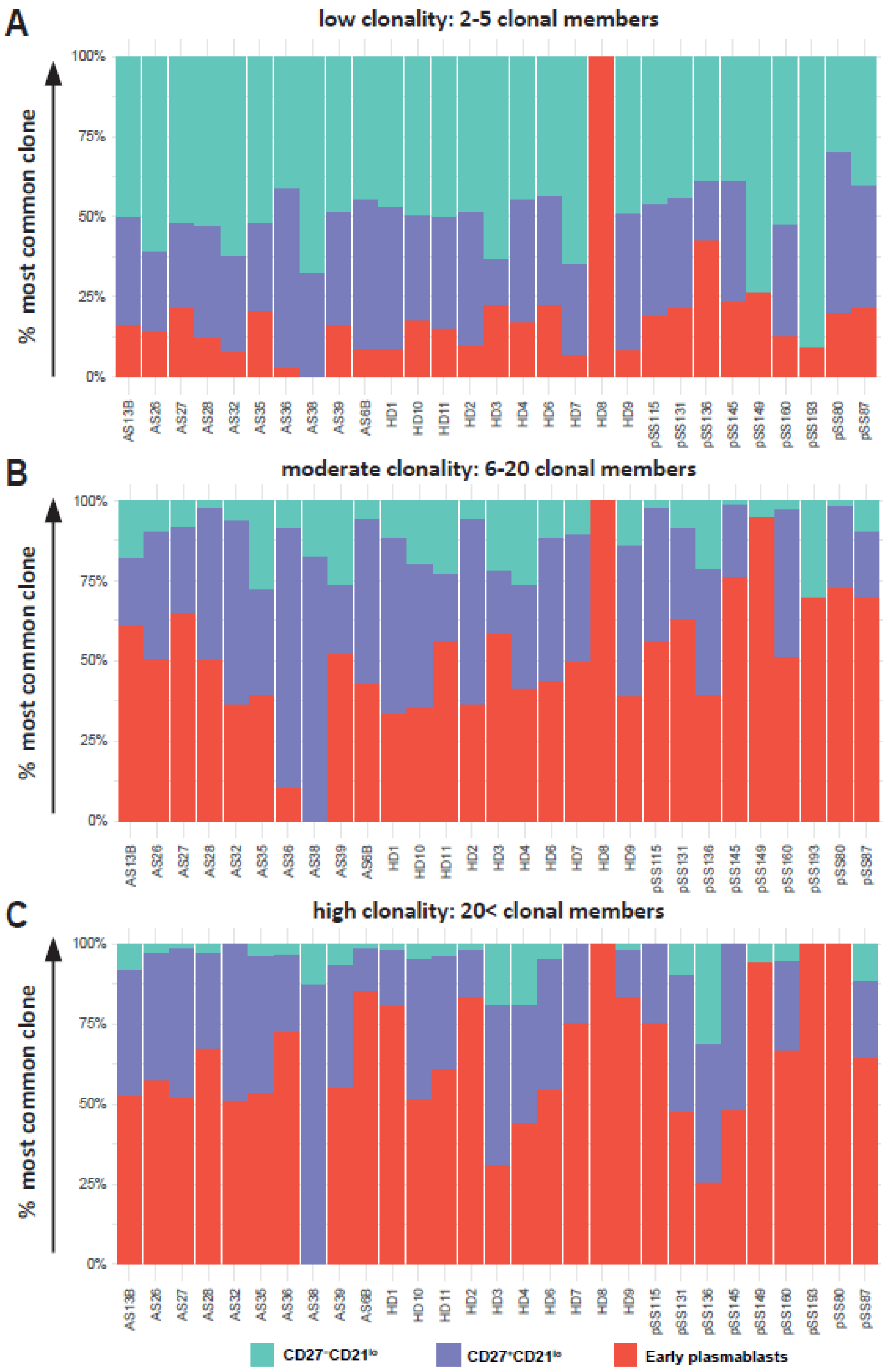

Supplementary Figure 2. Stacked-bar plots showing the percentage of the percentages of B cell populations (CD27$^-$CD21^{lo} B cells, CD27$^+$CD21^{lo} B cells and early plasmablasts) among clonal groups. Clonal groups were categorized as (**A**) low clonality (2-5 clonal members), (**B**) moderate clonality (6-20 clonal members), and (**C**) high clonality (20< clonal members) derived from clonally-related sequences of CD27$^-$CD21^{lo} B cells, CD27$^+$CD21^{lo} B cells and early PBs, shown for healthy controls (HC, N=10), Sjögren's disease (SjD, N=9), and radiographic axial spondyloarthritis (r-axSpA), N=10). Within these groups, clones were represented by the most common B cell population found among members in a clone. Afterwards, the fraction of representing B cell populations was calculated of the total clones.

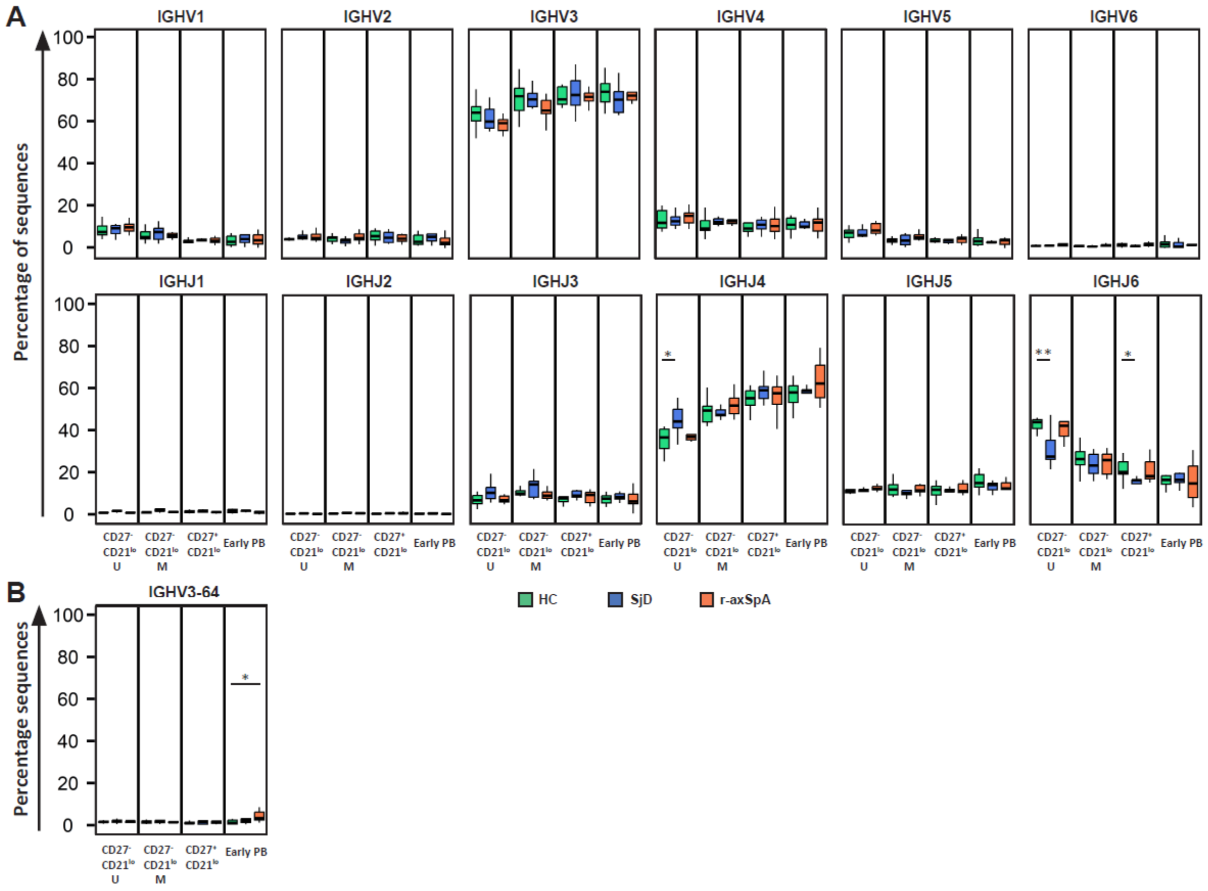

Supplementary Figure 3. (**A**) Boxplots depicting the usage of V and J family genes and (**B**) usage of the IGHV3-64 gene of unique sequences from $CD27^{-}CD21^{lo}$ B cells, $CD27^{+}CD21^{lo}$ B cells and early plasmablasts (PB) derived of healthy controls (HC, n=10), Sjögren's disease (SjD, n=9) and radiographic axial spondyloarthritis (r-axSpA, n=10) patients, as percentage of the total number of unique sequences. Boxplots showing the The boxplots are presented with horizontal lines indicating the medians, with two hinges displaying the 25th and 75th percentiles, and jitter points reflecting study participants. The unpaired Wilcoxon test was used to compare the HC reference group with the SjD and r-axSpA patient groups P-values <0.05 are considered significant. *<0.05, **<0.01.

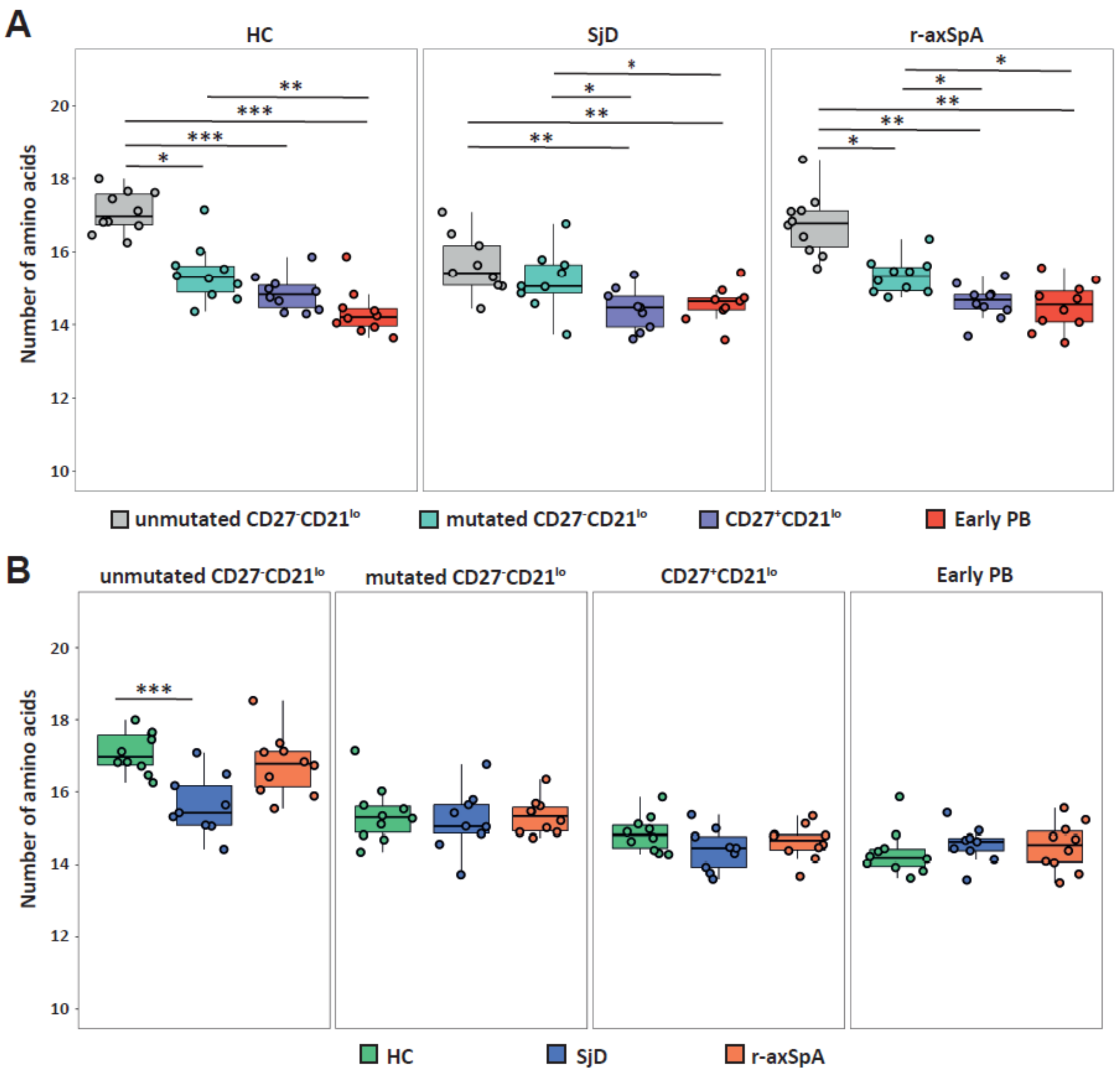

Supplementary Figure 4. The number of amino-acids comprising the CDR3 length was studied (**A**) among unmutated CD27$^-$CD21^{lo} B cells, mutated CD27$^-$CD21^{lo} B cells, CD27$^+$CD21^{lo} B cells and early plasmablasts (PB), between healthy controls (HC, n=10), patients with Sjögren's disease (SjD, n=9) and radiographic axial spondyloarthritis (r-axSpA, n=10). (**B**) The CDR3 amino-acid length is displayed for each study group between the four B cell subsets. Boxplots are presented with horizontal lines indicating the medians and include jitter points per subject. The Friedman test with Dunn's multiple comparisons correction was used to compare multiple B cell subsets within one study group. P-values <0.05 are considered significant. *<0.05, **<0.01, ***<0.001.

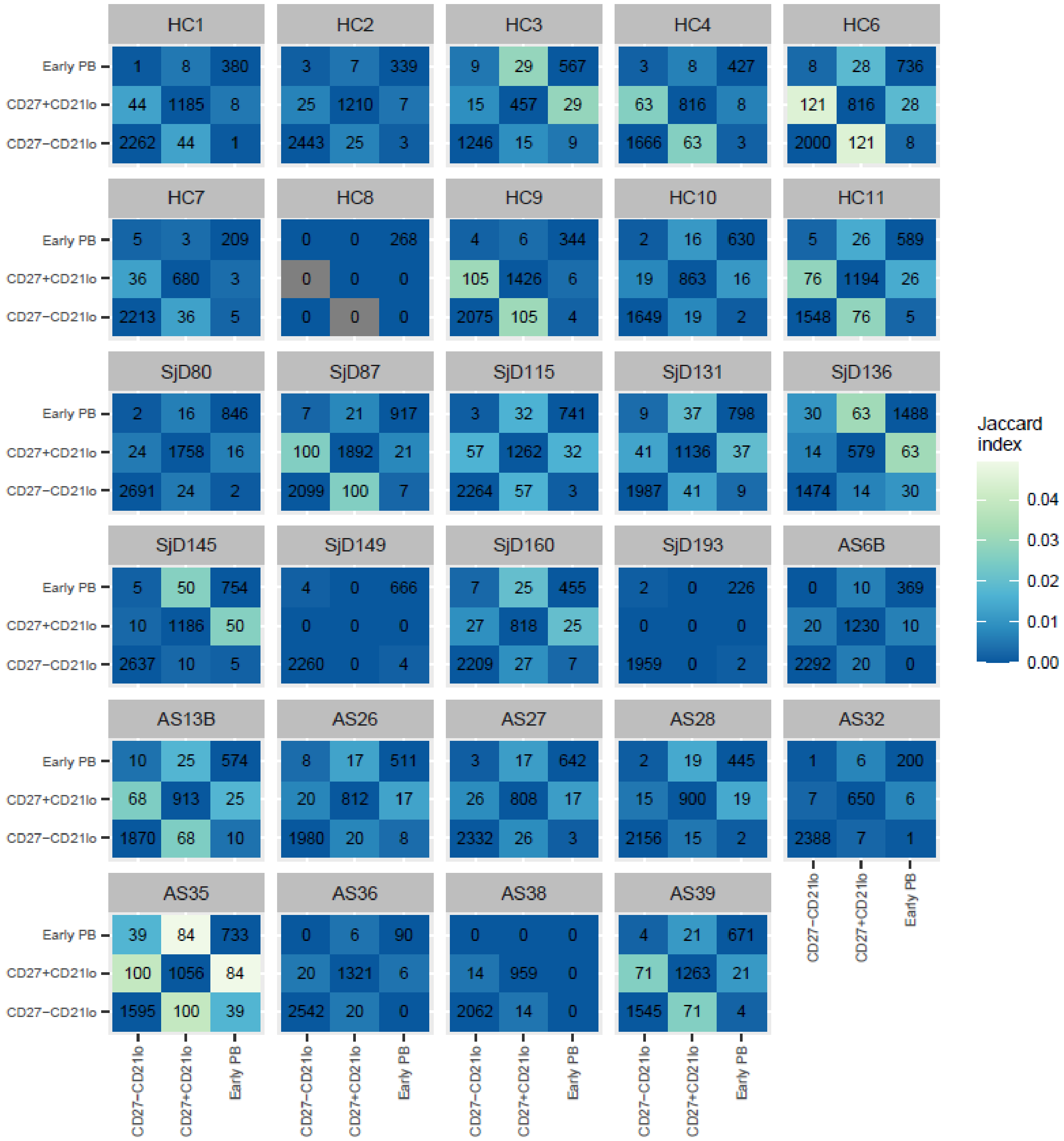

Supplementary Figure 5. Heatmaps illustrate the number of clones that share members among CD27^{-} CD21^{lo} B cells, CD27^{+}CD21^{lo} B cells and early plasmablasts (PB) for each study participant (n=29). Additionally, the Jaccard index is displayed for all combinations between these B cell populations.

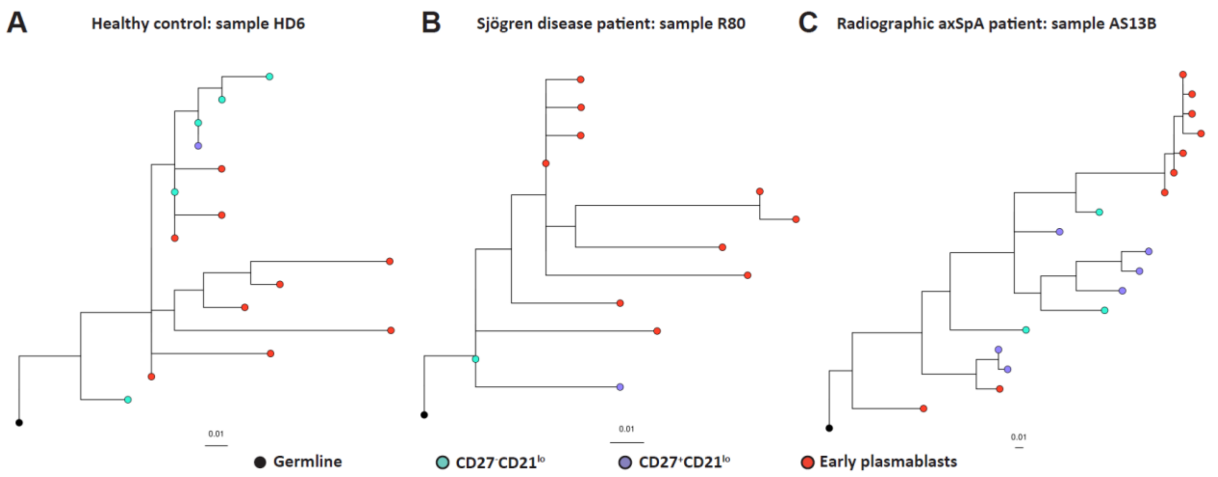

Supplementary Figure 6. Phylogenetic clonal lineage trees are shown for one healthy control, one Sjögren's disease patient, and one patient with radiographic axial spondyloarthritis (axSpA). Tips are labelled by B cell population: $CD27^{-}CD21^{lo}$ B cells, $CD27^{+}CD21^{lo}$ B cells and early plasmablasts (PB). Branch lengths indicate the rate of somatic mutations per site, as depicted by the common scale bar.
